# Violet-Blue Light Arrays at 405 Nanometers Exert Enhanced Antimicrobial Activity for Photodisinfection of Monomicrobial Nosocomial Biofilms

**DOI:** 10.1128/AEM.01346-19

**Published:** 2019-10-16

**Authors:** F. D. Halstead, M. A. Hadis, N. Marley, K. Brock, M. R. Milward, P. R. Cooper, B. Oppenheim, W. M. Palin

**Affiliations:** a Clinical Microbiology, Queen Elizabeth Hospital, University Hospitals Birmingham NHS Foundation Trust, Birmingham, United Kingdom; b NIHR Surgical Reconstruction and Microbiology Research Centre, Queen Elizabeth Hospital, Birmingham, United Kingdom; c Institute of Microbiology and Infection, University of Birmingham, Birmingham, United Kingdom; d School of Dentistry, Birmingham Dental Hospital and School of Dentistry, University of Birmingham, Birmingham, United Kingdom; e Cancer Research UK Clinical Trials Unit, University of Birmingham, Birmingham, United Kingdom; Rutgers, The State University of New Jersey

**Keywords:** phototherapy, decontamination, photodisinfection, wound decontamination

## Abstract

Light-emitting diodes (LEDs) demonstrate therapeutic effects for a range of biomedical applications, including photodisinfection. Bands of specific wavelengths (centered at 405 nm) are reported to be the most antimicrobial; however, there remains no consensus on the most effective irradiation parameters for optimal photodisinfection. The aim of this study was to assess decontamination efficiency by direct photodisinfection of monomicrobial biofilms using a violet-blue light (VBL) single-wavelength array (SWA) and multiwavelength array (MWA). Mature biofilms of nosocomial bacteria (Acinetobacter baumannii, Pseudomonas aeruginosa, Escherichia coli, and Staphylococcus aureus) were grown on 96-well polypropylene PCR plates. The biofilms were then exposed to VBL for 2,700 s (SWA) and 1,170 s (MWA) to deliver 0 to 670 J/cm^2^, and the antibacterial activity of VBL was assessed by comparing the seeding of the irradiated and the nonirradiated biofilms. Nonirradiated groups were used as controls. The VBL arrays were characterized optically (spectral irradiance and beam profile) and thermally. The SWA delivered 401-nm VBL and the MWA delivered between 379-nm and 452-nm VBL, albeit at different irradiances and with different beam profiles. In both arrays, the irradiated groups were exposed to increased temperatures compared to the nonirradiated controls. All bacterial isolates were susceptible to VBL and demonstrated reductions in the seeding of exposed biofilms compared with the nonirradiated controls. VBL at 405 nm exerted the most antimicrobial activity, exhibiting reductions in seeding of up to 94%. Decontamination efficiency is dependent on the irradiation parameters, bacterial species and strain, and experimental conditions. Controlled experiments that ameliorate the heating effects and improve the optical properties are required to optimize the dosing parameters to advance the successful clinical translation of this technology.

**IMPORTANCE** This study reports the efficacy of VBL and blue light (BL) and their antimicrobial activity against mature biofilms of a range of important nosocomial pathogens. While this study investigated the antibacterial activity of a range of wavelengths of between 375 and 450 nm and identified a specific wavelength region (∼405 nm) with increased antibacterial activity, decontamination was dependent on the bacterial species, strain, irradiation parameters, and experimental conditions. Further research with controlled experiments that ameliorate the heating effects and improve the optical properties are required to optimize the dosing parameters to advance the successful clinical translation of this technology.

## Abstract (precis)

This study reports the efficacy of VBL and blue light (BL) and their antimicrobial activity against mature biofilms of a range of important nosocomial pathogens. While this study investigated the antibacterial activity of a range of wavelengths of between 375 and 450 nm and identified a specific wavelength region (∼405 nm) with increased antibacterial activity, decontamination was dependent on the bacterial species, strain, irradiation parameters, and experimental conditions. Further research with controlled experiments that ameliorate the heating effects and improve the optical properties are required to optimize the dosing parameters to advance the successful clinical translation of this technology.

## INTRODUCTION

Light-based antimicrobial therapies involve the indirect use of light energy (photodynamic therapy [PDT]; by application of exogenously applied photosensitizers) or the direct use of light energy (photodisinfection without photosensitizers). PDT generally involves the use of longer wavelengths (usually, red to near infrared) that excite photosensitizers, which react with molecular oxygen to produce reactive oxygen species (ROS). Direct photodisinfection, however, with UV light (∼100 to 280 nm; UVC) is well-known to have substantially enhanced germicidal effects compared with photodisinfection with light with longer wavelengths (∼360 to 400 nm; within the UVA wave band) and violet-blue light (VBL; 400 to 420 nm), since DNA does not absorb the latter longer wavelengths of light (1, 2). Notably, the oxidative damage caused by UVA exposure is thought to be irreparable, whereas the effects of UVC can be overcome by innate cellular DNA repair mechanisms (3). Furthermore, UVC sterilization processes must be restricted since the risk of high-dose exposure presents severe health and safety concerns in humans. Longer-wavelength UVA and VBL reportedly target endogenous microbial photosensitizers (porphyrins) that react to produce ROS, which exert significant antimicrobial effects (4).

While the mechanisms of direct photoinduced bacterial killing have not been fully elucidated, it is proposed that the endogenous porphyrins (present in bacterial cell walls) are able to absorb light and transfer energy, leading to the production of highly cytotoxic ROS, such as peroxides, superoxide ions, and hydroxyl radicals (5) or singlet oxygen (^1^O_2_) (4, 6–8). The concentration-dependent antimicrobial effects of porphyrin have been reported in a number of studies, with low porphyrin concentrations limiting the antimicrobial activity of light, which can be reversed by inducing endogenous porphyrin production (9, 10). Interestingly, absorption by porphyrins (and the subsequent oxidative damage induced) may not be the sole cause of cell death (11), with reports indicating that VBL exposure is also able to upregulate the production of proteins by phage present in the bacteria, leading to bacterial death (12).

Porphyrins strongly absorb light at about 400 to 420 nm (known as the Soret band), with the peak absorbance occurring at approximately 405 nm (13). Consequently, 405-nm VBL has consistently been reported to be the most antimicrobial (4, 14, 15) and has been shown to inactivate many common bacterial pathogens, including Clostridium difficile, Escherichia coli, and methicillin-resistant Staphylococcus aureus (MRSA) (14–18). Reports also exist of its activity against mycobacteria (19) and fungi (20).

VBL is considered to be relatively ineffective at inducing DNA damage in humans and is reported to not be absorbed by endogenous DNA (220 to 300 nm; maximum λ, 254 nm) (1, 2). Consequently, without the need for exogenous chemicals and the reduced photon energy of longer wavelengths, direct photodisinfection using VBL may allow for the development of more convenient and safer antimicrobial protocols for patient treatment or applications involving patient- and practitioner-occupied settings.

Recently, devices emitting light at 405 nm have been incorporated into a new disinfection technology termed the high-intensity narrow-spectrum light environmental decontamination system (HINS-EDS) (21–24). The HINS-EDS is a ceiling-mounted LED array which delivers low-irradiance 405-nm light (irradiance, 0.1 to 0.5 mW/cm^2^) continuously to decontaminate surfaces in hospital operating theaters (16, 22). Evaluation studies showed that there was a statistically significant 91% reduction in the numbers of culturable *Staphylococcus* bacteria following 24 h of use in an unoccupied patient’s isolation room (21) and reductions of 56 to 86% when used in burns isolation rooms when an MRSA-positive patient was present during the treatment.

Environmental decontamination is an important method for reducing the threat from nosocomial pathogens and involves cleaning with detergents (to physically remove pathogens and organic matter) (25), followed by treatment with a low-level disinfectant to destroy any remaining bacteria (25, 26). This is laborious and relatively expensive and does not always eradicate the pathogens from the nosocomial environment (as evidenced by numerous reports of the transmission of nosocomial pathogens, despite terminal room cleaning [27]). We have previously shown in the *in vitro* setting that 405-nm VBL is effective at reducing the seeding of preformed mature biofilms of 34 nosocomial pathogens, including Acinetobacter baumannii, Enterobacter cloacae, Stenotrophomonas maltophilia, Pseudomonas aeruginosa, E. coli, Staphylococcus aureus, Enterococcus faecium, Klebsiella pneumoniae, and Elizabethkingia meningoseptica (28), and therefore, we propose that VBL provides a promising technology for disinfection of the nosocomial environment.

The aim of this study was to investigate if specific wavelength bands of VBL (375 nm to 420 nm) and blue light (BL; 420 nm to 450 nm) have antimicrobial activity against mature nosocomial biofilms of P. aeruginosa, A. baumannii, E. coli, and S. aureus. We chose to test mature, well-established biofilms, as the biofilm mode of growth represents the main mode of growth used by bacteria to survive in the nosocomial environment.

The following hypotheses were tested: (i) irrespective of irradiance, wavelengths of <405 nm and wavelengths of >405 nm within the VBL range would not result in a significant antimicrobial effect against any species or strain; (ii) irradiance (in milliwatts per square centimeter), exposure time (in seconds), and radiant exposure (irradiance × exposure time; in joules per square centimeter) would significantly influence the antimicrobial effects at 405 nm; and (iii) antimicrobial effects would be species and strain dependent.

## RESULTS

### Characterization. (i) Optical.

**FIG 1:**
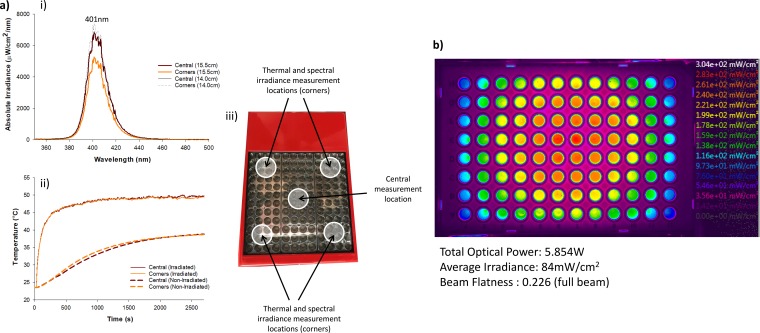
(a) Optical and thermal characterization of the SWA device showing the spectral irradiance (the measured peak wavelength is shown above the peak) (i), the corresponding temperature measurements (ii), and the location of the point measurements for spectral irradiance and temperature measurements with respect to the SWA device (iii). (b) The beam profile of the SWA device projected onto a 96-well peg plate (the distance from the array to the top of the cone is 14 cm) with a calibrated color scale on the right. While the full beam flatness is 0.226, within each well the beam is relatively homogeneous with a flatness ratio of >0.6. For each individual well, the irradiance ranges from 276 mW/cm^2^ (central wells) to 76 mW/cm^2^ (outer corner wells), with the average irradiance across the whole plate being 84 mW/cm^2^.

**FIG 2:**
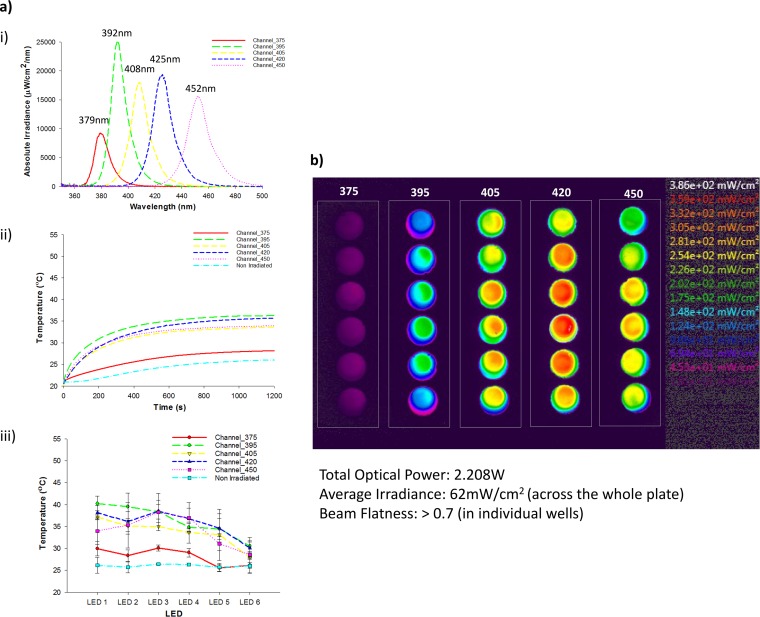
(a) Optical and thermal characterization of the MWA device showing the spectral irradiance for each channel (the measured peak wavelengths are reported above each spectrum) (i), the average temperature measurement in each channel over a 20-min exposure period (ii), and the maximum temperature for each LED (*n* = 3), with error bars representing standard deviations (the nonirradiated channel is measured in column 2 [no LEDs]) (iii). (b) The beam profile of the MWA device projected onto an Edmund Optics opal glass target screen from a distance of 5 mm (representative images are projected onto 96-well plate culture surfaces). Calibrated irradiance color scales are shown on the right. Each well represents a relatively homogeneous beam with a flatness ratio of >0.7. While an international filter was used to correct for the CCD sensor response at between 400 and 450 nm, wavelengths below 400 nm remain underrepresented and the beam images for those channels should be interpreted cautiously.

Spectral characterization of the arrays identified peak wavelengths for the single-wavelength array (SWA) at 401 nm (Fig. 1a) and multiwavelength array (MWA) between 379 nm and 452 nm (Fig. 2a). The 12-by-12 grid of reflectorized light-emitting diodes (LEDs) in the SWA produced a total power of 5.85 W (Fig. 1b), whereas 2.21 W was produced by the 6-by-5 grid of LEDs in the MWA (Fig. 2b), albeit with different wavelengths. The absolute irradiance revealed significant differences that were dependent on the light source, wavelength, and measurement location (*P* < 0.05). The average irradiance across the plate surfaces was significantly lower for the MWA than for the SWA (62 mW/cm^2^ and 84 mW/cm^2^, respectively; *P* < 0.05). For the SWA, the irradiance delivered was significantly higher in the center (*P* < 0.05) that at the edge (276 mW/cm^2^ to 76 mW/cm^2^, respectively). For the irradiated pegs, the irradiance ranged from 90 mW/cm^2^ to 248 mW/cm^2^, with the average being 190 ± 43.7 mW/cm^2^ (see the plate layout in Fig. S1 in the supplemental material).

The MWA exhibited significantly lower irradiance at 375 nm (114 ± 34 mW/cm^2^) than at the other wavelengths (356 ± 10 mW/cm^2^). The 405-nm channel specifically had irradiance ranging from 264 mW/cm^2^ to 430 mW/cm^2^ (average, 350 ± 56 mW/cm^2^), which was significantly higher than the irradiance delivered by the SWA at a similar wavelength (*P* < 0.05). Beam profile analysis confirmed a Gaussian beam distribution for the SWA (Fig. 1b), with a beam flatness ratio of 0.23 across the plate. However, within each well, the flatness ratio for the SWA was >0.6 and that for the MWA was >0.7.

### (ii) Thermal.

Significant exposure time-dependent thermal differences were measured for both arrays and were dependent on the light source and wavelength (Fig. 1a and 2a; *P* < 0.05). The SWA produced uniform thermal increases across the plate irrespective of location (central location or corners; Fig. 1a; *P* > 0.05). The maximum temperature exhibited during SWA irradiation was significantly higher for the irradiated groups than for the nonirradiated control groups (50.0 ± 0.2°C and 39.3 ± 0.2°C, respectively; *P* < 0.05). Similarly, the maximum temperature for the nonirradiated groups for the MWA was significantly lower than that for the irradiated groups (26.1 ± 0.3°C and 33.6 ± 3.2°C, respectively; *P* < 0.05; Fig. 2a). However, for the MWA, a temperature gradient existed in each channel between the first and the last LED, but the maximum temperature during irradiation was always <40°C (Fig. 2a).

### Response of biofilm isolates to multiple wavelengths of VBL delivered by the MWA.

The minimum biofilm eradication concentration (MBEC) assay showed that all bacterial species and strains were susceptible to at least one wavelength of VBL from the MWA, as demonstrated by reductions in seeding and/or biofilm biomass (for the results of the statistical analyses, see Tables 1 and 2). Additionally, all bacteria were susceptible to the VBL from the SWA platform, with the reductions in seeding (compared to that for an untreated control) ranging from 43.6 to 94.3% and with concurrent reductions in biofilm biomass occurring following exposure (thereby implying antibiofilm effects).

**TABLE 1:**
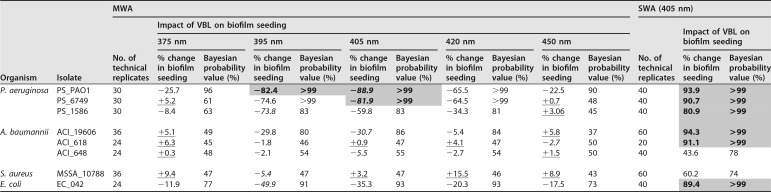
Impact of BL and VBL treatment (delivered by both platforms) on biofilm seeding for the range of isolates included in the test panel^*a*^

a Mean average values are provided for the percent reduction (denoted by minus signs) or increase (denoted by plus signs) in biofilm seeding. Underlining refers to wavelengths that resulted in increased biofilm seeding, and italics indicate the wavelength that resulted in the largest reduction for that isolate. Gray shading and boldface denote results where there was greater than or equal to an 80% reduction in seeding, with Bayesian probability represented as a percentage.

**TABLE 2:**
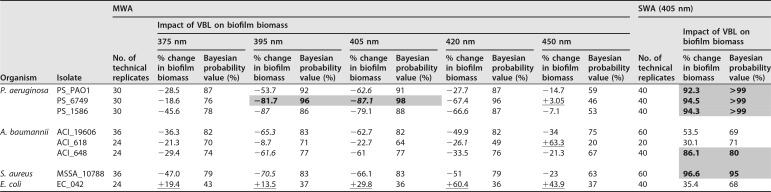
Impact of the BL and VBL treatment (delivered by both platforms) on biofilm biomass for the range of isolates included in the test panel^*a*^

a Mean average values are provided for the percent reduction (denoted by minus signs) or increase (denoted by plus signs) in biofilm biomass. Underlining refers to wavelengths that resulted in increased biofilm biomass, and italics indicate the wavelength that resulted in the largest reduction for that isolate. Gray shading and boldface denote results where there was greater than or equal to an 80% reduction in biomass, with Bayesian probability represented as a percentage.

### (i) Impacts on biofilm seeding.

Reductions in biofilm seeding were observed for all isolates when the biofilms were exposed to VBL of five different wavelengths using the MWA (Table 1). These are indicated in Table 1, with gray shading and boldface representing reductions in seeding of at least 80% and italic text denoting the most favorable result for that isolate across all wavelengths of the MWA.

The percent reductions in biofilm seeding (of the test peg compared with a nonexposed control peg) ranged from 1.8% (with strain ACI_618 at 395 nm) to 88.9% (with strain PS_PAO1 at 405 nm). The P. aeruginosa isolates were the most susceptible to VBL, with reductions in seeding of the exposed biofilms ranging from 73.8% (for strain PS_1586, a clinical burn wound isolate, at 395 nm) to 88.9% (for PS_PAO1, a control strain, at 405 nm). According to our Bayesian analysis, we can be 99% certain that (for PS_PAO1) the effect of the treatment was superior to that of no treatment (positive control), contingent on the model form chosen and the data that we have observed. Smaller reductions in seeding occurred across all the wavelengths for the A. baumannii isolates. Treatment of ACI_19606 led to a 30.7% reduction in seeding at 405 nm (probability value, 86%), and there were increases in seeding or very marginal reductions (of 2.7 and 5.5%) for the other two A. baumannii isolates at a range of wavelengths (e.g., for ACI_618, there was reduced seeding at 395 and 420 nm and increased seeding at the other wavelengths). For methicillin-susceptible S. aureus (MSSA) strain MSSA_10788, 395 nm was the only wavelength resulting in reduced seeding (of 5.4%), with increased seeding being observed for all other wavelengths. Furthermore, there were only marginal reductions in biofilm seeding for the single E. coli isolate tested (EC_042), with a maximum reduction of 49.9% being recorded at 395 nm.

**FIG 3:**
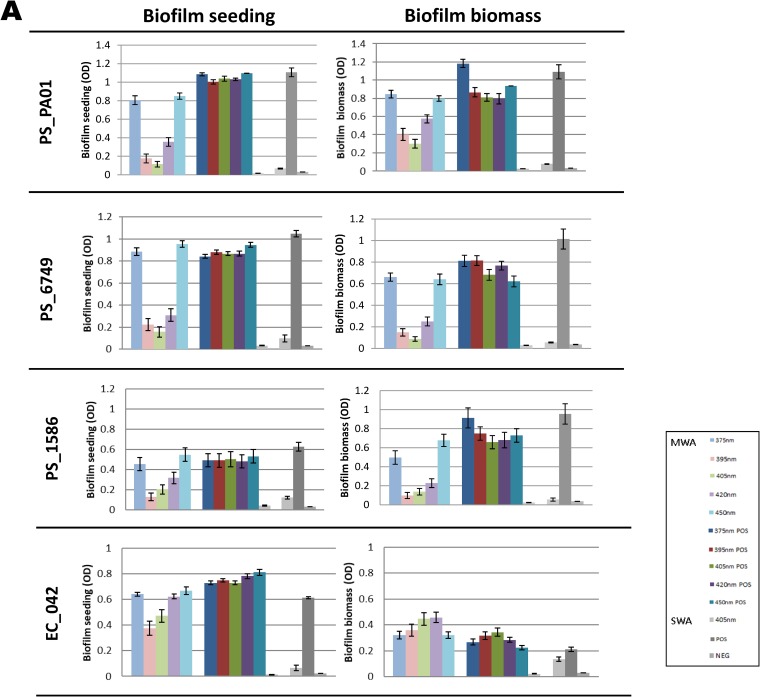

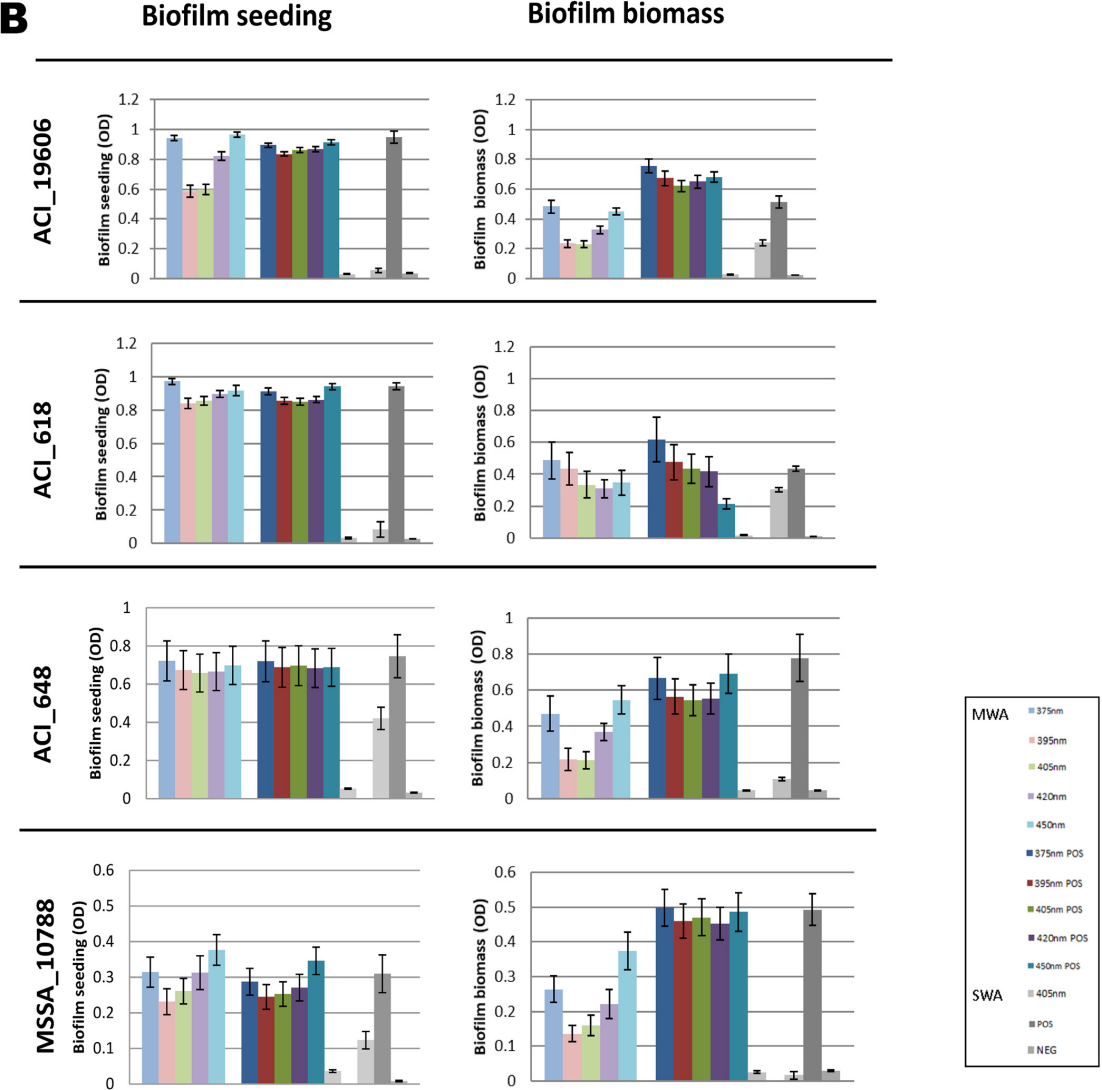
(A) Graphs showing the effect of VBL exposure of the biofilms in terms of the effects on both biofilm seeding and biofilm biomass for strains PS_PAO1, PS_6749, PS_1586, and EC-042. On each graph, biofilm seeding or biomass is shown on the *y* axis, with each bar showing the effect of a particular treatment, which is indicated in the key. The error bars represent the standard error. The durations of exposure for the MWA and SWA were 19 min 30 s (19.5 min) and 45 min, respectively. (B) Graphs showing the effect of the VBL exposure of the biofilms in terms of the effects on both biofilm seeding and biofilm biomass for ACI_19606, ACI_618, ACI_648, and MSSA_10788. On each graph, biofilm seeding or biomass is shown on the *y* axis, with each bar showing the effect of a particular treatment, which is indicated in the key. The error bars represent the standard error. The durations of exposure for the MWA and SWA were 19 min 30 s (19.5 min) and 45 min, respectively.

For all isolates, the three middle wavelengths (395, 405, and 420 nm) of VBL were the most antimicrobial, with the largest reductions in seeding occurring at these wavelengths for seven of the eight isolates (all isolates except ACI_618, where 450 nm led to the greatest reductions). In the majority of cases, the two other wavelengths of VBL (375 and 450 nm) were associated with either very small reductions in biofilm seeding (PS_PAO1 and EC_042) or small increases in seeding ranging from 0.3% to 9.4%. The results are underlined in Table 1. Figures 3A and B summarize the results for all isolates and for each wavelength of VBL.

### (ii) Impacts on biofilm biomass.

Reductions in biofilm biomass (as measured by solubilizing crystal violet [CV] and measuring the resulting optical density [OD]) were recorded for seven of the eight isolates (for all isolates except EC_042) and for all wavelengths of VBL (Table 2). The highest values for reduction ranged from 26.1% to 87.1%, and good reductions (of 65.3, 26.1, and 61.6%) were also apparent for the three A. baumannii isolates (ACI_19606, ACI_618, and ACI_648, respectively), where there had been poor impacts of the treatment on biofilm seeding (Fig. 3B). There were no reductions in biofilm biomass following treatment for the single E. coli isolate (EC_042). For this isolate, treatment prompted the isolate to increase biofilm production at all wavelengths (as evidenced by an increased biofilm biomass) (Fig. 3A). As for the biofilm seeding data, the 395- and 405-nm wavelengths were associated with the greatest antibiofilm effects.

### Sensitivity of biofilm isolates to 405-nm VBL delivered by the SWA. (i) Impacts on biofilm seeding.

All of the isolates were susceptible to killing by the 400- to 405-nm light delivered by the SWA, with the reductions in seeding being greater than 80% for six of the eight isolates (Table 1). In contrast to the MVA results, A. baumannii ACI_19606 was the most susceptible (reduction, 94.3%) and ACI_648 was the least susceptible (reduction, 43.6%). The Bayesian analysis performed returned probability values of greater than 99% for all isolates (except MSSA_10788), and therefore, with 99% certainty we can conclude that the effect of the treatment is superior to that of no treatment (positive control), again contingent on the model form chosen and the data that we have observed.

### (ii) Impacts on biofilm biomass.

Reductions in biofilm biomass were apparent for all isolates with the VBL delivered by the SWA. These ranged from 30.1% (ACI_618) to 96.6% (MSSA_10788). Interestingly, there was no obvious correlation of these results with the biofilm seeding results. Generally, the isolates with the highest reductions in biofilm seeding experienced the lowest reductions in biofilm biomass, and vice versa (Tables 1 and 2).

### Comparison of 395- and 405-nm VBL results from both platforms.

The results for the 395-nm and 405-nm VBL delivered from the MWA and the 401-nm VBL delivered from the SWA were compared owing to similarities in the wavelengths. For all isolates and for both the seeding and the biomass data sets, the reductions were higher following the SWA treatment than following the MWA treatment (Tables 1 and 2). There were also discrepancies, especially for the A. baumannii isolates, where there were reductions in seeding of 94.3, 91.1, and 43.6% with the SWA (for ACI_19606, ACI_618, and ACI_648, respectively), whereas with the MVA, the percent changes to biofilm seeding were −29.8, −1.8, and −2.1%, respectively (at 395 nm), and −30.7, +0.9, and −5.5%, respectively (at 405 nm), for the same isolates.

## DISCUSSION

VBL treatment of biofilms of all isolates at various wavelengths of between 375 nm and 420 nm was successful in terms of antibacterial action, resulting in reduced seeding of treated/exposed biofilms compared with that for a nonirradiated control. Thus, the first hypothesis was rejected. The radiant exposure significantly influenced the antimicrobial effects, and thus, the second hypothesis was accepted. From the small number of isolates tested, these effects were also species and strain dependent, with P. aeruginosa being the most susceptible and A. baumannii being the least. Therefore, the third hypothesis was also accepted. In terms of susceptibility, further research is required to establish the reasons for these differences, which may be related to the differences in porphyrins (such as structure and/or concentration) or a secondary photosensitizing mechanism owing to the pigments produced by *Pseudomonas* spp. compared with A. baumannii.

In agreement with previous research, the most effective wavelength range for antimicrobial effects in this study was 375 nm to 420 nm, with the greatest antimicrobial effects being observed at wavelengths of 395 nm and 405 nm. This was somewhat as expected, since these wavelengths coincide with the Soret band for absorption (where absorbance peaks at 403 nm), commonly reported in endogenous bacterial porphyrins (13). Wavelengths outside of this range (375 nm, 420 nm, and 450 nm) are most likely not absorbed by endogenous porphyrins and are therefore unlikely to generate ROS through Soret band absorption. While narrow band, the polychromatic 375-nm and 420-nm LEDs with a maximum transmission peak outside the Soret band still emitted wavelengths within in it (albeit with less spectral overlap) (Fig. 2a, panel i). Indeed, the reduced spectral irradiance and radiant exposure at 375 nm [due to UV absorption by the poly(methyl methacrylate) lens] may have also reduced the antimicrobial effects compared with those achieved at other wavelengths. This assumes no effect of photon energy (the number of photons delivered), which is higher at 375 nm, since a similar irradiance has a higher photon energy at shorter wavelengths. While it is possible to standardize the number of photons delivered, the aim of this study was to identify the wavelength bands that may be antimicrobial, and thus, the standardized irradiance for all LEDs (except the 375-nm LED) was a suitable comparator and similar to what was used in previous photobiological research that has explored the effect of wavelength on cellular processes (29).

It was interesting to observe that the majority of the bacteria irradiated with 375-nm and 450-nm LEDs exhibited increases in biofilm seeding. This seems logical, since these wavelengths are unlikely to be absorbed by the Soret band contained within the porphyrins, and hence, the antimicrobial effects seen with 395 and 405 nm are likely absent or reduced. The growth and increased seeding of these biofilms may be explained through (non-Soret band) absorption of the light, resulting in the generation of heat and the stimulation of bacterial growth owing to heat. Indeed, heating may also produce small amounts of ROS, which may also be beneficial for bacterial growth (30). Increases in biofilm seeding have previously been reported by Halstead et al. (28), who found one isolate of Enterobacter cloacae with enhanced biofilm seeding, and various other authors, who have found that if the dose is not optimal, BL and VBL can promote biofilm formation (31, 32). This would clearly not be a favorable outcome were this technology used clinically, and therefore, further research is warranted.

At 375 and 450 nm, there were two isolates (E. coli isolate EC_042 and P. aeruginosa isolate PS-PAO1) with which reductions in biofilm seeding were observed. There is no obvious difference between these two isolates and the others in terms of pigmentation, and so it would be interesting to explore the reasons for the difference in antimicrobial activity in future work.

While the SWA delivered wavelengths similar to the 405-nm channel of the MWA, spectroradiometric and thermal analyses revealed significant optical and thermal differences. First, the SWA consists of a 12-by-12 grid of reflectorized LEDs that produce a wide footprint across multiple wells, whereas the MWA consists of individual surface-mount-device LEDs designed to focus inside a single well. The total power output of the SWA device was ∼6 W, whereas that for the individual LEDs in the MWA, which is delivered through a lens, was <50 mW (total power, ∼2.2 W). The SWA device is operated from a distance of 15.5 cm (in line with our previous research [28]) and provides irradiation directly onto the culture plates or pegs from above. Since each LED produces a divergent beam which is reflected by the design of the optics, the beam produced at a distance of 15.5 cm exhibits a Gaussian profile, with the highest irradiance being delivered in the center of the beam. Furthermore, light that was delivered from the SWA through the plastic cultureware was reflected from the aluminum base.

However, the individual LED and lens combination in the MWA produced a more uniform irradiance per well (Fig. 2b; flatness ratio, >0.7). The MWA delivers light through the base of the 96-well plate from a distance of 5 mm, and for the 405-nm channel specifically, irradiance ranges from 264 mW/cm^2^ to 430 mW/cm^2^ (average, 350 ± 56 mW/cm^2^). Therefore, the SWA delivered higher radiant exposures ranging from 243 J/cm^2^ to 670 J/cm^2^ (average, 513 ± 118 J/cm^2^), based on the irradiance values delivered to culture wells. The MWA delivered less irradiation (range, 309 J/cm^2^ to 503 J/cm^2^; average, 409 ± 66 J/cm^2^), since the exposure time for this device was reduced to 19 min 30 s. Furthermore, due to surface reflection, those radiant exposure values for the SWA are likely to be significantly higher.

The platforms also differ, in that the SWA operates in a semienclosed compartment (the top surface is open) with minimal airflow, whereas the MWA is in a fully enclosed compartment in which directional airflow is provided using fans and heat sinks. Consequently, the SWA exposes bacteria to a significantly higher temperature over a longer period of time than the MWA (Fig. 2). These high temperatures and prolonged heat exposures can reportedly denature DNA and may have a significant impact on bacterial viability through thermal denaturing of proteins and DNA (33). It could also be possible that higher temperature can increase the absorption coefficient of porphyrins, thus lowering the threshold for photosensitization (34, 35). Further research is warranted to investigate the possible additive and synergistic effects of light and temperature (at levels noninjurious to human tissue).

Despite these encouraging *in vitro* results, there are several limitations of the study which should be acknowledged. First, the study involved only a small number of bacterial isolates, which were used to form monomicrobial biofilms on plastic surfaces. This is clearly an oversimplification of biofilm formation *in vivo*, and future work should involve polymicrobial biofilms for translation toward clinical decontamination applications. Second, an absolute measure of the change in bacterial loads following treatment in terms of log counts per milliliter was not calculated to assess the viability of treated biofilms through growth assays. While this is important to assess the impact of disinfectants, the current measurement method provided a higher throughput to screen the effects of multiple wavelengths on numerous species and intraspecies strains. Log reduction calculations will also be used in future studies, as we develop our understanding of the biological mechanisms associated with photodisinfection and optimize species-dependent dose parameters. Finally, the experiments were not controlled for the impact of heating on bacterial viability. Indeed, the SWA results in an ∼50°C increase in temperature at the site where biofilms are grown following the 45 min of treatment, which is likely to also have some antimicrobial effects. While such temperature increases may limit clinical applications *in vivo*, further work is warranted to investigate these effects with irradiation parameters that dissipate heating effects to achieve low irradiation temperatures which are, ideally, maintained at body temperature.

In conclusion, this study reports the efficacy of VBL and BL and their antimicrobial activity against mature biofilms of a range of important nosocomial pathogens. While this study investigated the antibacterial activity of a range of wavelengths of between 375 to 450 nm and identified a specific wavelength region (∼405 nm) with increased antibacterial activity, decontamination was dependent on the bacterial species, strain, irradiation parameters, and experimental conditions. Further research with controlled experiments that ameliorate heating effects and improve optical properties is required to optimize the dosing parameters to advance the successful clinical translation of this technology.

## MATERIALS AND METHODS

### LED array characterization. (i) LED arrays.

Two platforms were used to deliver VBL to the mature bacterial biofilms: an in-house-manufactured multiwavelength array (MWA) and a commercially produced single-wavelength array (SWA) platform (Loctite; Henkel-Loctite, Hemel Hempstead, UK).

**FIG 4:**
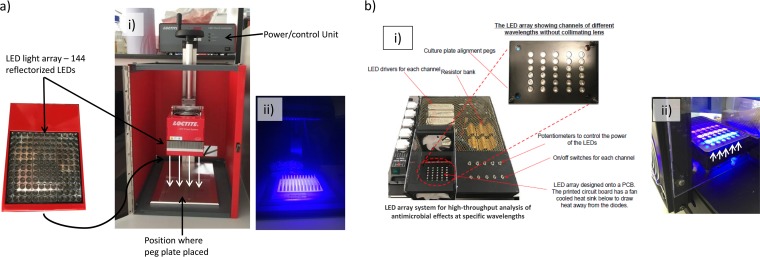
The two different platforms used for the delivery of VBL. (a) The single-wavelength array (SWA), showing the 144-reflectorized-LED array (405 nm) and the direction and location of irradiation (i) and the array in use (ii). (b) The multiwavelength array (MWA), showing the platform and how it is constructed (i) and the array in use (ii). White arrows denote the direction of VBL delivery. PCB, printed circuit board.

The SWA platform (Fig. 4a) produced a single peak of VBL through a 144-reflectorized-LED flood array and for all experiments was operated from an array base distance of 15.5 cm. The MWA was designed and manufactured in-house based on previous work by the authors (29) for use in the high-throughput analysis of the antibacterial effects of five specific wavelength bands of VBL (Fig. 4b). The array was designed to fit a 96-well microtiter tray (MTT) and consisted of five channels of different VBL wavelengths (375 nm, 395 nm, 405 nm, 420 nm, and 450 nm) which were calibrated to deliver an irradiance of approximately 114 mW/cm^2^ (375 nm) or 348 to 369 mW/cm^2^ (all other wavelengths) at the base of the 96-well plates (Table 3).

**TABLE 3:** Wavelengths of VBL produced by platforms alongside their irradiance, exposure time, and dose

Platform	Wavelength (nm)	Avg spectral irradiance (mW/cm^2^) of LEDs ± SD	Exposure time (s)^*a*^	Avg dose (J/cm^2^) delivered to exposed pegs^*b*^
MWA	375	114 ± 34	1,170	133.4
	395	358 ± 23	1,170	418.9
	405	350 ± 56	1,170	409.5
	420	369 ± 18	1,170	431.7
	450	348 ± 37	1,170	407.2
SWA	405	190 ± 43.7	2,700	513.0

a The exposure time corresponds to 19 min 30 s for MWA and 45 min (for SWA).

b Where dose = average irradiance (in watts) multiplied by exposure time (in seconds). Plate layouts of the exposed pegs are shown in Fig. S1 in the supplemental material.

### (ii) Spectral irradiance.

A miniature spectrometer (model STS-UV-RAD; Ocean Optics, UK) with a CC-3-DA Spectralon cosine corrector (Ocean Optics, UK) was used to measure the spectral irradiance of the SWA device. The spectrometer was radiometrically calibrated in compliance with National Institute of Standards and Technology (NIST) practices recommended in *NIST Handbook 150-2E* (36) and allowed spectral irradiance measurements to be made over a relatively large collection area (diameter = 7.14 mm, outer diameter = 12.7 mm). A fiber-based UV-visible spectrometer (model USB4000; Ocean Optics, UK) was used to assess the absolute spectral irradiance for the MWA. The spectrometer arrangement consisted of a 200-μm optical fiber and an opaline glass CC3 cosine corrector (diameter of collection area = 3.90 mm, outer diameter = 6.35 mm; Ocean Optics, UK) which was calibrated in-house to NIST standards against a traceable light source (model Mikropack DH2000; Ocean Optics, UK).

For the MWA device, a black 96-well plate (Corning; Sigma-Aldrich) was placed into the array, and the irradiance delivered to the base of the plate was measured by inserting the cosine corrector end of the fiber into each well before taking the readings. As the wells of the 96-well plate had diameters of ∼6.5 mm, this allowed reliable and concentric alignment with each of the adjacent LEDs. For the SWA device, the cosine corrector was directed and placed parallel to the light-emitting surface at a distance of 15.5 cm. Spectral irradiance measurements (*n* = 3) were made using OceanView software (Ocean Optics, UK) for each LED of the MWA and centrally and 25 mm from each corner for the SWA. The absolute irradiance was determined by integration of the spectral irradiance within the LED emission region.

### (iii) Beam profilometry.

Beam profile images were recorded using a silicon-based charge-coupled-device (CCD) camera beam profiler (model SP620; Ophir, Spiricon, Israel). The camera utilized a 50-mm closed-circuit television (CCTV) lens (Ophir, Spiricon, Israel) that was focused onto a diffusing opal glass target screen (Edmund Optics, UK) held at a 5-mm LED-target screen distance for the MWA or onto a 96-well peg plate at a distance of 15.5 cm (from the array to the base of the peg) for the SWA. The maximum CCD sensor area was utilized by enlarging the beam image projected on the sensor by using a combination of spacer rings (20 mm; Ophir, Spiricon) between the lens and the CCD sensor. A linear scaling calibration was performed to enable pixel dimension calibration in the plane of the targets. For each measurement, a combination of neutral-density filters (Ophir, Spiricon, Israel) was used to avoid CCD sensor saturation. In addition, for the MWA, an international corrective filter (International Light Technologies, Peabody, MA, USA) was used to correct for the spectral response of the CCD sensor. Prior to beam imaging in each measurement, the system was corrected for ambient light and pixel response using the UltraCal function in Beam Gage software (Ophir, Spiricon, Israel). The diameter of the active light beam (D4σ or the second moment width; ISO reference 11145 3.5.2 [37]) was determined automatically by the Beam Gage software on the basis of the linear calibration. Predetermined power values, calculated from the spectral irradiance of the MWA (the product of the irradiance and the sensor area) or measured using a 10-mm-diameter photodiode sensor (PD300 photodiode; Ophir, Spiricon) held at a 15.5-cm distance for the SWA, were used for optical calibration of the images. The power distribution over the active beam area (irradiance) was then determined as the inputted power divided by the corresponding collection area. The total power, beam flatness, and average irradiance for each device was determined using a digital aperture within the software.

### (iv) Temperature measurements.

The temperature at the culture surfaces was measured using K-type thermocouples (diameter, 1.21 mm; Maplin, UK) and a multichannel data logger (*n* = 8; model TC-08, Pico, UK) by simulated irradiations and recorded using PicoLog software (Pico, UK) in order to mimic the temperature change in the biofilm. For the MWA, sterile black 96-microwell plates were used, with the thermocouples being inserted through the lids, secured directly above each LED, and placed in contact with the base of the plates. For the SWA, thermocouples were inserted into white 96-well peg plates centrally (*n* = 4) and on the outer wells (*n* = 4) and held 14 cm away from the light source (the distance between the peg tip and the array). The temperature was measured every second for up to 1 h.

### Biofilm assays. (i) Bacterial strains.

A series of *in vitro* experiments was conducted with a panel of organisms (Table 4) to determine the efficacy of the different wavelengths (375 to 450 nm) of VBL against bacteria persisting in a biofilm (attached to a surface) mode of growth.

**TABLE 4:** Clinical and control strains used in the study^*a*^

Study identifier	Organism	Description
ACI_618	Acinetobacter baumannii	QEHB clinical outbreak isolate
ACI_648	Acinetobacter baumannii	QEHB clinical outbreak isolate
ACI_19606	Acinetobacter baumannii	ATCC 19606 (unique)
PS_PAO1	Pseudomonas aeruginosa	ATCC 15692
PS_6749	Pseudomonas aeruginosa	NCTC 6749
PS_1586	Pseudomonas aeruginosa	QEHB clinical burn isolate
EC_042	Escherichia coli	EAEC 042
MSSA_10788	Staphylococcus aureus	NCTC 10788

a ATCC, American Type Culture Collection; NCTC, National Collection of Type Cultures; EAEC, enteroaggregative Escherichia coli; QEHB, Queen Elizabeth Hospital.

The panel comprised control and clinical nosocomial isolates and consisted of strains of P. aeruginosa (*n* = 3), A. baumannii (*n* = 3), E. coli (*n* = 1), and S. aureus (*n* = 1) which had previously been reported to form biofilms (28, 38, 39). From a decontamination point of view, A. baumannii was included, as it is a key nosocomial pathogen which survives in hospital and health care environments, despite conditions such as desiccation, nutrient starvation, and antimicrobial chemicals (e.g., disinfectants) (40, 41). Isolates of E. coli (EC_042) and S. aureus (NCTC 10788; which is a recognized test strain in the European (EN) standards of the British Standards Institution, e.g., standard EN 13727 [42] for assessing the efficacy of chemical disinfectants) were included as comparators. All isolates were stored at −80°C on Protect beads and were routinely cultured on cysteine lactose electrolyte-deficient (CLED) or blood agar prior to each experiment. There was no ongoing maintenance of the test organisms in the stock cultures.

### (ii) Biofilm growth standardization.

The antibacterial activity of VBL against preformed biofilms was assessed by conducting minimum biofilm eradication concentration (MBEC) experiments (43) on each isolate. Overnight cultures of the test strains were made by inoculating three to five colonies into 5 ml of fresh Luria broth (LB; Sigma-Aldrich, UK) and incubating at 37°C in O_2_ for 18 to 24 h. The inocula were diluted in fresh antibiotic-free Mueller-Hinton (MH) broth to an optical density at 600 nm (OD_600_) of 0.1 (which corresponds to approximately 1 × 10^5^ CFU/ml), and then 50 μl was seeded into the wells of a 96-well microtiter tray (MTT). A positive control (50 μl of organisms diluted to an OD_600_ of 0.1) and a negative control (50 μl MH broth) were included per blue light time point and wavelength to be tested.

To produce a transferable biofilm, a 96-well polypropylene PCR plate (Starlabs, UK) was then placed into the MTT so that each well contained a peg on which biofilms could form, before the plates were sealed and statically incubated at 33°C in O_2_ for 72 h. After 72 h, the pegs were removed and washed in an MTT containing sterile water (to remove any unbound cells). The test pegs (which contained a biofilm if they were placed into the test or control wells of the MTT or which were devoid of biofilm if they were in the negative-control [broth-only] wells) were then exposed to light from either the MWA or the SWA as detailed below, while a second group of similar pegs was not irradiated within the same plates. Bacterial peg plates were exposed to both light sources in the same experiment, thereby restricting the only changing variable to the light source. The peg plate layouts are shown in Fig. S1 in the supplemental material.

### Biofilm irradiation. (i) Multiple-wavelength array.

For MWA exposure, the plates in which biofilms were attached to pegs were placed into an optically transparent (within the visible region [29]) polystyrene black plastic plate carrier. Nonirradiated control pegs were achieved by blocking the light path between the carrier plate and the pegs using several layers of light-insulating tape. The plate carrier was then positioned directly above the MWA so that the pegs were concentrically and reproducibly aligned with the adjacent LEDs. Light was delivered through the base of the carrier plate to the biofilm-attached pegs using five channels of VBL and BL (375 nm to 450 nm) for 19 min 30 s delivering a radiant exposure of ∼133.4 J/cm^2^ (375 nm), 418.9 J/cm^2^ (395 nm), 409.5 J/cm^2^ (405 nm), 431.7 J/cm^2^ (420 nm), 407.2 J/cm^2^ (450 nm), or 0 J/cm^2^ (nonirradiated control groups).

### (ii) Single-wavelength array.

For exposure using the SWA platform, the test peg plate was placed onto the metal base plate of the array with the pegs facing upwards (with a distance of 15.5 cm between the light source and the metal base plate and 14 cm between the light source and the tops of the pegs). The positive-control (bacteria only) and negative-control (sterile broth only) peg plates were placed in a clean, empty MTT and wrapped in foil. Following this, the peg plates were directly exposed to VBL for 45 min (corresponding to an average VBL dose of 513 J/cm^2^ [range, 243 to 670 J/cm^2^; standard deviation, 44.2 J/cm^2^] to the exposed test pegs). The foil placed around the control plate prevented the pegs from receiving any blue light treatment (and, hence, these positive-control biofilms were not exposed to the blue light).

### (iii) MBEC and crystal violet assay.

Following irradiation, the pegs were carefully placed into an MTT containing 50 μl sterile MH broth (herein referred to as “reporter broth”) for overnight incubation. After 18 h, the OD of the reporter broth was measured using a spectrophotometer (model 6300 spectrophotometer; Jenway, UK), which allowed us to assess the approximate amount of planktonic cells that had been released from the biofilms following VBL exposure or no exposure (as in the controls). This is referred to as “seeding.”

To demonstrate the presence of biofilms on the pegs, crystal violet (CV) assays were additionally performed on the pegs after the OD of the reporter broth had been measured. Subsequently, the pegs were placed into MTTs containing 50 μl of 1% CV (which binds to any microbial biomass of biofilm present), followed by washing (to remove unbound CV) and subsequent solubilization of the CV in 50 μl of 70% ethanol. The peg biofilm biomass was then measured using OD readings as described above, and the presence of the biofilm was confirmed. Two biological replicates and 12 to 18 technical replicates (summarized in Table 1) were performed for each strain and VBL wavelength, respectively.

### Statistical analysis.

LED array characterization data were analyzed using multifactorial analysis of variance (ANOVA) using a general linear model (GLM) ANOVA and subsequent *post hoc* Tukey comparisons (with 95% confidence levels) using Minitab (version 15) software.

Bayesian hierarchical regression models were used to analyze other data sets. The response variable was the difference in seeding between treatment 1 (i.e., MWA- or SWA-treated pegs) and treatment 2 (i.e., the positive control) with an otherwise matched plate position, wavelength, organism, and experimental iteration. We used group-level intercepts and gradient terms with respect to wavelength and its square, with grouping being performed at the organism and experiment level. That is, our analysis model assumed that each organism and each experiment were associated with coefficients that were exchangeable draws from the normal distributions of effects. This method facilitated the analysis of all data in a single model, incorporating terms for putative explanatory factors like wavelength while reflecting within-group correlations at the organism and experiment level. The method does not assume that replicates are independent.

We used a unit normal prior on the population-level intercept and generalized Student’s *t* test distributions for the group-level standard deviation terms, with zero location parameter, scale parameter 1, and 3 degrees of freedom. These were chosen to be regularizing, allowing a wide range of parameter values while ruling out implausibly large values. For instance, all the response outcome values that we observed were contained within the range (−1.5, 0.5). We did not know what form that the analysis model would take *a priori*.

During the model-building process, we discarded simpler models that did not use quadratic wavelength effects or assumed simpler group structures. These were discarded on the basis of the poor fits implied by the posterior predictive distributions and the leave-one-out information criterion (44, 45). We also discarded more complex models. Cubic effects in wavelength were found to add little to the model fit. The models were fitted using the Stan program (46) and the brms package in R (47, 48). The chains that we used for inference were fitted with zero divergent transitions in postwarmup sampling, yielding R-hat values of (1, 1.01) and large effective sample sizes. Model checks showed that prediction errors appeared to be independent of the wavelength and the levels of the predictions themselves (i.e., larger predictions were not associated with larger errors). Furthermore, the errors and group-level terms showed symmetry and a central tendency, with normal distributions being valid working models. Posterior samples were arranged using the tidybayes package in R (49) and visualized using the ggplot2 program (50).

## Supplementary Material

Supplemental file 1
