# Supplementary material for: Violet-Blue Light Arrays at 405 Nanometers Exert Enhanced Antimicrobial Activity for Photodisinfection of Monomicrobial Nosocomial Biofilms
Source: Appl Environ Microbiol. 2019 Oct 16;85(21):e01346-19. doi: 10.1128/AEM.01346-19 (PMC6803304; doi:10.1128/AEM.01346-19)
Supplement: Supplemental file 1 [file AEM.01346-19-s0001.pdf]

Panel A: SWA

i) Layout of the test pegs

	1	2	3	4	5	6	7	8	9	10	11	12	**TEST PEGS**
A													
B	T	T	T	T	T	T	T	T	T	T			Organism A
C	T	T	T	T	T	T	T	T	T	T			Organism A
D													
E													
F	T	T	T	T	T	T	T	T	T	T			Organism B
G	T	T	T	T	T	T	T	T	T	T			Organism B
H													

2 bacterial isolates per plate (as per the colours)

ii) Layout of the control pegs

	1	2	3	4	5	6	7	8	9	10	11	12	**CONTROL PLATE**
A													
B	PC	PC	PC	PC	PC	PC	NC	NC	NC	NC			Organism A
C	PC	PC	PC	PC	PC	PC	NC	NC	NC	NC			Organism A
D							NC	NC	NC	NC			
E							NC	NC	NC	NC			
F	PC	PC	PC	PC	PC	PC	NC	NC	NC	NC			Organism B
G	PC	PC	PC	PC	PC	PC	NC	NC	NC	NC			Organism B
H													

Panel B: MWA

i) Layout of the test pegs

<table>
<tr><th></th><th>1</th><th>2</th><th>3</th><th>4</th><th>5</th><th>6</th><th>7</th><th>8</th><th>9</th><th>10</th><th>11</th><th>12</th><th>TEST PEGS</th></tr>
<tr><td>A</td><td></td><td></td><td></td><td></td><td></td><td></td><td></td><td></td><td></td><td></td><td></td><td></td><td>1 organism per plate</td></tr>
<tr><td>B</td><td></td><td></td><td rowspan="6">375</td><td></td><td rowspan="6">395</td><td></td><td rowspan="6">405</td><td></td><td rowspan="6">420</td><td></td><td rowspan="6">450</td><td></td><td></td></tr>
<tr><td>C</td><td></td><td></td><td></td><td></td><td></td><td></td><td></td><td></td><td></td></tr>
<tr><td>D</td><td></td><td></td><td></td><td></td><td></td><td></td><td></td><td></td><td></td></tr>
<tr><td>E</td><td></td><td></td><td></td><td></td><td></td><td></td><td></td><td></td><td></td></tr>
<tr><td>F</td><td></td><td></td><td></td><td></td><td></td><td></td><td></td><td></td><td></td></tr>
<tr><td>G</td><td></td><td></td><td></td><td></td><td></td><td></td><td></td><td></td><td></td></tr>
<tr><td>H</td><td></td><td></td><td></td><td></td><td></td><td></td><td></td><td></td><td></td><td></td><td></td><td></td><td></td></tr>
</table>

ii) Layout of the control pegs

<table>
<tr><th></th><th>1</th><th>2</th><th>3</th><th>4</th><th>5</th><th>6</th><th>7</th><th>8</th><th>9</th><th>10</th><th>11</th><th>12</th><th>CONTROL PLATE</th></tr>
<tr><td>A</td><td></td><td></td><td></td><td></td><td></td><td></td><td></td><td></td><td></td><td></td><td></td><td></td><td></td></tr>
<tr><td>B</td><td></td><td rowspan="6">Negative control</td><td rowspan="6">Positive control</td><td rowspan="6">Negative control</td><td rowspan="6">Positive control</td><td rowspan="6">Negative control</td><td rowspan="6">Positive control</td><td rowspan="6">Negative control</td><td rowspan="6">Positive control</td><td rowspan="6">Negative control</td><td rowspan="6">Positive control</td><td></td><td></td></tr>
<tr><td>C</td><td></td><td></td><td></td></tr>
<tr><td>D</td><td></td><td></td><td></td></tr>
<tr><td>E</td><td></td><td></td><td></td></tr>
<tr><td>F</td><td></td><td></td><td></td></tr>
<tr><td>G</td><td></td><td></td><td></td></tr>
<tr><td>H</td><td></td><td></td><td></td><td></td><td></td><td></td><td></td><td></td><td></td><td></td><td></td><td></td><td></td></tr>
</table>

Supplementary Figure 1: Showing the layout of the test and control pegs for both the SWA (Panel A), and the MWA (Panel B), in terms of i) layout of the test pegs (with test pegs denoted by 'T'), and ii) the layout of the control pegs (with 'PC' denoting positive control, and 'NC' denoting negative control).
